# Giant cell tumor along with secondary aneurysmal bone cyst of scapula: A rare presentation

**DOI:** 10.4103/0973-6042.42579

**Published:** 2008

**Authors:** Rana K. Sherwani, Sufian Zaheer, Amir B. Sabir, Siddharth Goel

**Affiliations:** Department of Pathology, JN Medical College, AMU, Aligarh-202 002, UP, India; 1 Department of Orthopedic Surgery, JN Medical College, AMU, Aligarh-202 002, UP, India

**Keywords:** Aneurysmal bone cyst, giant cell tumor, scapula

## Abstract

Giant cell tumor (GCT) is a distinctive lesion characterized by the proliferation of multinucleate giant cells in a stroma of mononuclear cells; it is generally seen in skeletally mature individuals. GCT of bone is usually found in the long bones around the knee or in the distal radius of young adults and is unusual in the flat bones. We report a case of GCT of the acromion of the scapula, with a secondary aneurysmal bone cyst, in a 30-year-old female. Based on our review of the English language medical literature, it appears that the occurrence of a GCT along with a secondary aneurysmal bone cyst in flat bones (e.g.. the scapula) is very rare.

## INTRODUCTION

Cooper first reported giant cell tumors (GCT) in the 18^th^ century; in 1940, Jaffe and Lichtenstein defined GCT more rigorously to distinguish it from other tumors.[1]

GCT of bone is an uncommon lesion representing between 4 and 9.5% of primary bone neoplasms and is thought to originate from undifferentiated cells of the supporting tissues of bone marrow. It is most commonly seen in early adulthood, with a peak incidence in the third decade and with a slight female preponderance; it is usually seen in the skeletally mature patient. The tumor is most commonly located around the knee, with the distal radius being the next most common site.[2] Flat bone involvement is rare. GCT usually occur de novo but may also occur as a rare complication of Paget's disease of the bone.[3] In a review of English language medical literature in 1989, Aoki found only 13 cases of GCT of the scapula and only three of these cases involved the acromion.[4] Isolated involvement of the acromion of the scapula by GCT, along with an associated aneurysmal bone cyst, is a rare presentation.

## CASE REPORT

**Figure 1:**
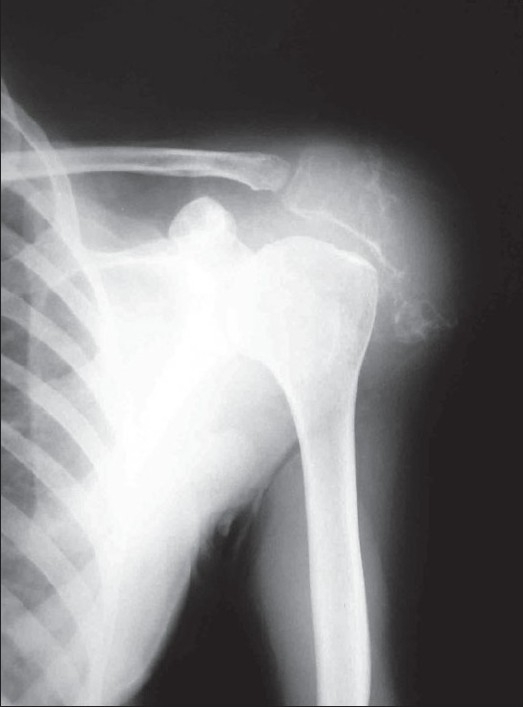
X-ray shows a lytic lesion with a soap bubble appearance in the acromion of the right scapula, with destruction of the periosteum and a prominent overlying soft tissue shadow

A 30-year-old female presented to us with swelling of the right shoulder since 1 year, along with the complaints of restricted mobility of the right arm. On local examination, a globular swelling was present at the right shoulder, measuring 6 × 4.5 cm and having a cystic to firm consistency. There were dilated veins over the swelling and the local temperature was raised. X-ray of the right shoulder showed an expansile lytic lesion with soap bubble appearance of the lateral part of the acromion of the right scapula; there was destruction of periosteum and a prominent overlying soft tissue shadow [Figure 1]. Magnetic resonance imaging showed a large heterogeneous mass in the acromion. Serum chemistry, including serum calcium level, phosphorus, alkaline phosphatase, and parathormone level, was within the normal range. Renal and liver function tests were also within the normal range

**Figure 2:**
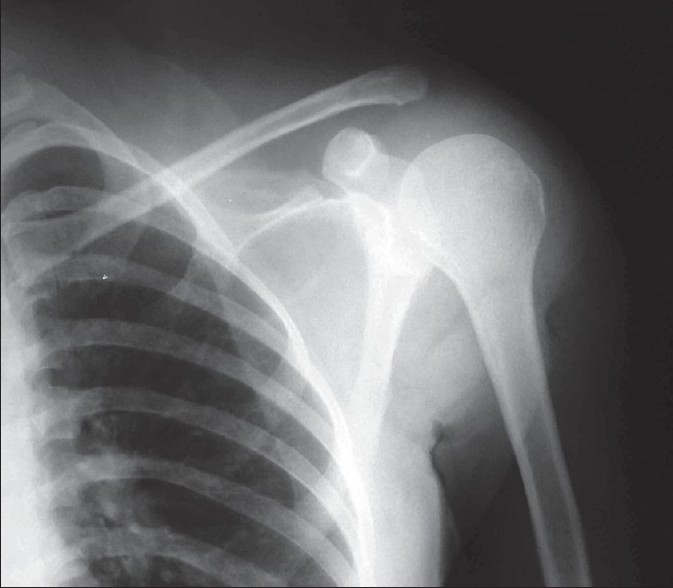
Postoperative x-ray of the patient

The patient underwent a partial scapulectomy with removal of the acromion and spine of scapula [Figure 2]. The tissue was submitted for histopathological examination.

Gross examination revealed irregular tissue pieces, with firm to hard areas measuring 6 × 4.5 × 4 cm. It had a heterogeneous appearance, with creamy areas mixed with hemorrhagic areas. The cut surface had a variegated appearance, with cystic, hemorrhagic, and necrotic areas.

**Figure 3:**
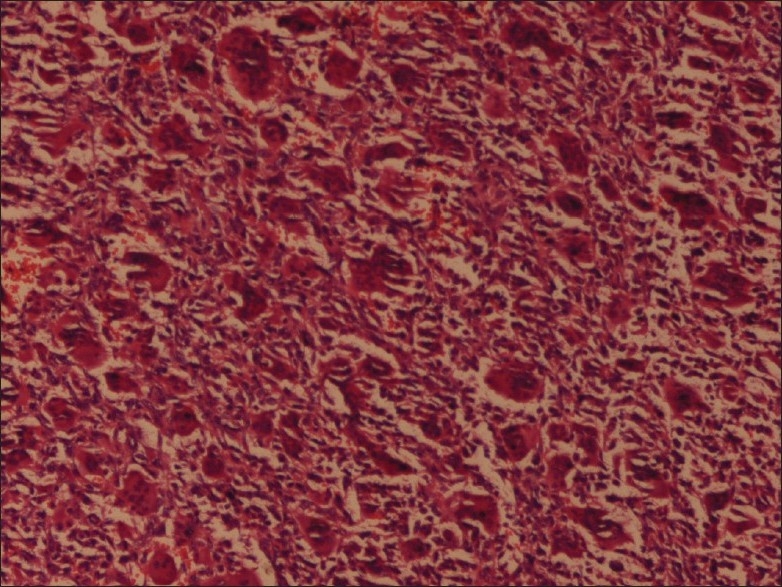
Pictomicrograph of a giant cell tumor showing polygonal to elongated mononuclear cells mixed with numerous osteoclast-like giant cells (H and E; 400×)

**Figure 4:**
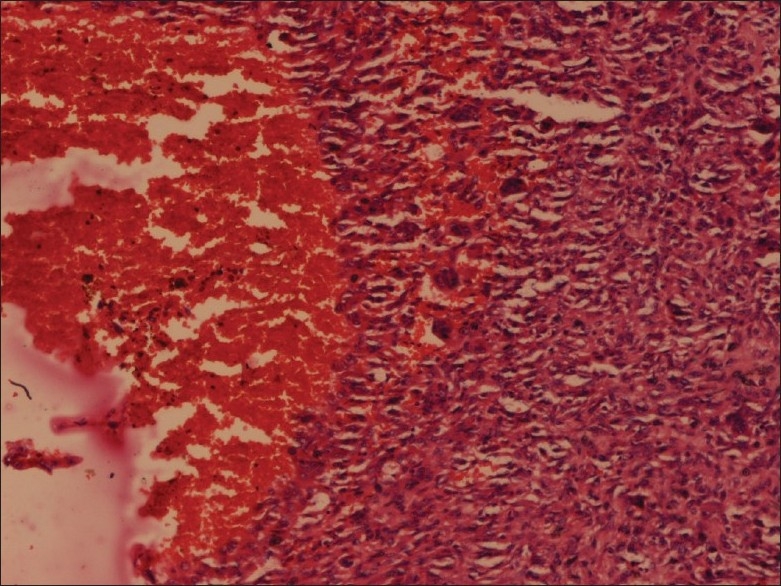
Foci of secondary aneurysmal bone cyst (H and E; 400×)

Microscopic examination showed features consistent with the diagnosis of GCT, with polygonal to elongated mononuclear cells mixed with numerous osteoclast-like giant cells [Figure 3]. In some areas, morphology consistent with aneurysmal bone cyst was noted [Figure 4]. A peripheral shell of reactive bone was present. No evidence of malignant change was seen.

Five months post surgery the patient is doing well. Early active exercises were started for the elbow and hand and passive and active movements of the right shoulder were encouraged. Following this, the patient was able to use his elbow and hand normally and the abduction is now 100° at the operated shoulder joint.

## DISCUSSION

GCT of the bone, previously known as myeloid sarcoma, tumor of myeloplaxus, osteoblastoclastoma, and osteoclastoma,[5] has a distinctive microscopic appearance, and its diagnosis is usually not difficult despite the fact that the gross appearance of a GCT is less characteristic than its microscopic appearance. The tumor is usually seen as a soft, brown mass within which areas of hemorrhage, which appear dark red, and areas of collagen, which appear gray, may be observed.

Although Cooper first reported GCT in the 18^th^ century, it was in 1940 that Jaffe and Lichtenstein[1] described GCT in detail to distinguish it from other tumors. Although many different types of bony neoplasms may contain giant cells, GCT has giant cells as its most prominent component. The lesion almost always involves the epiphysis or the ends of long bones. In long bones, GCT is considered an epiphyseal lesion. A diagnosis other than GCT, such as an osteosarcoma rich in giant cells or an aneurysmal bone cyst, must be considered if a lesion containing giant cells is seen in the metaphysis or the diaphysis of a long bone.[2]

GCT usually occurs after completion of maturation of the skeleton. More than 80% of the patients with GCT are between 20–40 years of age.[5] About 75–90% of GCTs occurs in long tubular bone. More than 50% of GCTs arise in the distal femur and the proximal tibia. Other common sites of involvement include the distal radius and the sacrum. Less common sites include flat bones like ribs, skull, patella, sternum, and clavicle.[2,6] The rarity of the occurrence of GCT at these sites may often lead to misdiagnosis, both clinically and radiologically; however, if a giant cell lesion is seen in the flat bones it is important to rule out hyperparathyroidism and giant cell-rich osteosarcoma before making the diagnosis of GCT.[2] Distinction from hyperparathyroidism can be easily made on the basis of serum calcium, phosphate, alkaline phosphatase, and parathormone levels. However, true GCT in the flat bones have been well documented in literature.[5] True GCT of the small bones of the hands and feet do occur but, at these sites, the more likely diagnosis is that of aneurysmal bone cyst.[2]

Involvement of the scapula by a GCT has only been rarely reported in the English language medical literature. Windeyer and Woodyat[7] described a GCT of the scapula in 1949. Samilson and Tuli *et al.* described one case each of GCT of the scapula.[5] In 1989, Aoki *et al.*[4] reviewed 13 GCTs of the scapula and found only three cases in the acromion. In 1991, Park *et al.*[6] reported the first case of GCT of the scapula occurring in association with a secondary aneurysmal bone cyst in the English language medical literature.

On roentgenogram, a characteristic, purely lytic lesion with destruction is seen extending to the end of the bone. Mineralization within the lesion is lacking. The center is most radiolucent with increasing density towards the periphery. These tumors often present with cortical thinning, and may expand into the soft tissues surrounding the bone, or they may expand the bone extensively, remaining within an eggshell-thin rim of periosteal new bone.[5]

Histopathologically, there is proliferation of two cell populations. There are numerous multinucleate giant cells with, on an average, more than 50 nuclei. Intermixed with them are mononuclear cells, which are of uniform size and round to oval in shape. The nuclei of the giant cells resemble those of the mononuclear cells. There is no cytologic atypia and atypical mitotic figures are absent in a typical GCT. GCTs may show secondary aneurysmal bone cyst-like changes.[2]

We have reported this case with the purpose of emphasizing that GCT of flat bones, especially of the scapula, is very rare and the diagnosis may be missed, both clinically and radiologically, unless there is a high index of suspicion. On the other hand, the diagnosis of other conditions, e.g. hyperparathyroidism, must be ruled out satisfactorily before making the diagnosis of GCT by serum chemistry and parathormone level. What made our case even more worth reporting is the fact that the involvement of the acromial process of the scapula occurring in association with a secondary aneurysmal bone cyst has not been reported before.
